# Clinicians’ Perceptions of Challenges and Strategies of Transition from Assertive Community Treatment to Less Intensive Services

**DOI:** 10.1007/s10597-014-9706-y

**Published:** 2014-02-14

**Authors:** Molly T. Finnerty, Jennifer I. Manuel, Ana Z. Tochterman, Candice Stellato, Linda H. Fraser, Cecily A. S. Reber, Hima B. Reddy, Angela D. Miracle

**Affiliations:** 1 Department of Child and Adolescent Psychiatry/Bureau of Evidence Based Services and Implementation Science, New York University/New York State Office of Mental Health, 1051 Riverside Drive, Unit 104, New York, NY 10032 USA; 2 School of Social Work, Virginia Commonwealth University, 1001 West Franklin Street, P.O. Box 842027, Richmond, VA 3284-2027 USA; 3 New York State Psychiatric Institute, New York State Office of Mental Health, 1051 Riverside Drive, Unit 104, New York, NY 10032 USA; 4 Office of Coordinated Mental Health Services, New York City Department of Health and Mental Hygiene, 42-09 28th St., 19th floor CN No. 17, Long Island City, New York, NY 11101-4132 USA; 5 PGSP-Stanford Psy.D. Consortium, 1791 Arastradero Rd., Palo Alto, CA 94304 USA; 6 Fairleigh Dickinson University, 1000 River Road, Teaneck, NJ USA

**Keywords:** Assertive Community Treatment, Mental illness, Transition, Barriers to discharge, Facilitators of discharge

## Abstract

The study aimed to identify clinical strategies and challenges around transition from Assertive Community Treatment (ACT) to less intensive services. Six focus groups were conducted with ACT team leaders (n = 49). Themes were grouped under four intervention-focused domains: (1) client/clinical, (2) family and natural supports, (3) ACT staff and team, and (4) public mental health system. Barriers to transition included beliefs that clients and families would not want to terminate services (due to loss of relationships, fear of failure, preference for ACT model), clinical concerns that transition would not be successful (due to limited client skills, relapse without ACT support), systems challenges (clinic waiting lists, transportation barriers, eligibility restrictions, stigma against ACT clients), and staff ambivalence (loss of relationship with client, impact on caseload). Strategies to support transition included building skills for transition, engaging supports, celebrating success, enhanced coordination with new providers, and integrating and structuring transition in ACT routines.

## Introduction

Assertive Community Treatment (ACT) is the highest intensity service that can be received in the outpatient setting (Stein and Test 1980). ACT has a low caseload ratio, maintains a 24-h responsibility of care for clients, and delivers in vivo services in the community via a multidisciplinary team, which serves as the primary provider for clients. ACT is a client-centered, comprehensive mental health program that provides all psychiatric outpatient treatment, rehabilitation and support services to persons with severe mental illness, who are prone to frequent relapses and rehospitalizations, and who have severe psychosocial impairment. Numerous reviews have documented evidence for the efficacy of the ACT model, including reduced hospital use and increased housing stability (Bond et al. 2001; Mueser et al. 1998).

ACT was originally conceived as a time-unlimited service when it was developed more than three decades ago. Indeed, a “no discharge policy” is one measure of a high fidelity team (Teague et al. 1998). The original recommendation that ACT services should be time-unlimited was based on the initial ACT study that showed a decline in clinical gains 12 months after a planned termination of ACT services (Stein and Test 1980). Other studies found that in the absence of ACT services, clients’ mental health deteriorated (Fairweather et al. 1969; McRae et al. 1990). These studies and others provided evidence for time-unlimited treatment with persons diagnosed with severe mental illness. “Time-unlimited” services have been interpreted as “lifelong” services by many. However, the concept of providing intensive lifelong services to clients does not comport with longitudinal studies indicating that more than 50 % of clients recover over time (DeSisto et al. 1995; Harding et al. 1987; Strauss et al. 1985). These results challenge long held beliefs concerning the poor prognosis for individuals with severe mental illness, as well as the need for ACT for life.

The understanding of ACT as a lifelong service may have contributed to the underdevelopment of research on transition from ACT. However, a handful of studies suggest that ACT clients can transition successfully to lower levels of care. Rosenheck and Dennis (2001) found that clients could be selectively discharged from an ACT program for homeless individuals with serious mental illness, to lower levels of care without the loss of gains achieved from the ACT program. Subsequently, Rosenheck et al. (2010) reported that clients who were transitioned to less intensive services experienced greater clinical improvements, and had a higher quality of family relationships and overall higher quality of life compared to those who were not transitioned. Among those who transitioned, about 6 % returned to higher intensity services. In a retrospective case review, Hackman and Stowell (2009) examined outcomes of 67 individuals who were discharged from ACT to lower levels of service within the University of Maryland medical system. The majority of these clients (48 of 67) remained in less intensive services after an average follow-up period of 40 months. Chen and Herman (2012) conducted interviews and focus groups to examine ACT practitioners’ perspectives on discharge from ACT. Clinicians were divided in their beliefs about the role of ACT in recovery, while some ACT staff saw ACT as having an ongoing role to ensure sustained recovery, other staff saw clients transitioning to lower levels of service as part of recovery.

Taken together, published studies suggest that discharge from ACT marks a vulnerable period, during which there is a potential for deterioration and relapse, but where most clients selected for a planned discharge can successfully transition to a lower level of service. However, little is known about the clinical processes and strategies for promoting successful transition from ACT to less intensive services. Transition raises critical concerns about how to ensure that clients have support and a safety net during the transition period, highlighting the need for research in this area. This study aims to understand transition processes by delineating ACT team leaders’ perspectives on the challenges and strategies of transition from ACT to less intensive services. This project informs a statewide initiative of the New York State Office of Mental Health (OMH) to support transition from ACT to less intensive services.

## Methods

### Setting

New York State (NYS) operates 79 ACT teams in five regions (Western, Central, Hudson River Valley, Long Island, New York City), serving almost 5,000 persons who are diagnosed with severe mental illness and whose needs have not been adequately met by more traditional mental health services. ACT services are multidisciplinary with focus on assertive outreach, frequent contacts, flexible services, 24-7 coverage, community integration, and integrated health and mental health services (Center for Mental Health Services, Substance Abuse and Mental Health Services Administration 2008). Staffing ratios are low (1:10), teams provide a comprehensive set of other evidence-based treatments (e.g., Supported Employment, Integrated Dual Disorder Treatment, Family Consultation, and Wellness Self-Management), rehabilitation, case management, and support services.

### Sample

ACT team leaders were recruited for the study using purposive sampling techniques to elucidate processes across sites and regions. Coordinators from OMH field offices in each region distributed study information sheets to ACT team leaders, who were invited to participate in a focus group discussion in their region. Team leaders were exclusively included for three main reasons: (1) to ease recruitment as they attend monthly meetings at local OMH regional offices; (2) to avoid participants’ potential concerns around sharing responses among supervisors or subordinates, and (3) all team leaders on ACT teams have direct clinical contact with clients, and are responsible for clinical supervision of all team members, and consequently have a broad perspective on clinical strategies and issues for their team. A total of six focus group discussions were held between June and August of 2008. One focus group was held in each region and an additional focus group held in New York City, the largest region. Participants were 49 ACT team leaders, or 62 % of ACT team leaders across NYS, with an average of 8–9 participants in each regional focus group. All participants had a master’s degree or higher and represented a variety of disciplines (e.g., social work, nursing, counseling, psychiatry, and psychology). Participants had worked on their ACT teams between 2 and 8 years.

### Data Collection

We used focus groups to generate data in an effort to understand the nature of experiences of ACT service providers, and conceptualize the clinical strategies and challenges relevant to transition work. Each focus group discussion lasted approximately 90 minutes and was held in a private room at the OMH field office in each region. All focus groups were conducted using a semi-structured format guided by a protocol of questions and probes developed by the research team based on prior research on transition from ACT (Rosenheck and Dennis 2001; Salyers et al. 1998).The discussion guide focused on four general areas, (1) overview of ACT team services/clients and provider role; (2) current discharge practices and step-down/graduation approaches (What do you think makes discharge successful or unsuccessful?); (3) perceptions of core components/ingredients of step-down/graduation approaches; (4) general perceptions of step-down/graduation approach. Questions under each area were open-ended and designed to elicit a broad range of views and opinions from participants. Each group was led by two facilitators trained in qualitative methods. The lead facilitator had extensive training and experience in conducting focus groups in community mental health settings, and the co-facilitator had expertise in managing and evaluating ACT services.

### Analysis

Audio recordings of the focus group discussions were transcribed and then analyzed using a thematic analysis approach (Strauss and Corbin 1990). The development of the codes combined deductive (based on a priori categories, i.e., transition challenge, or transition strategy) and inductive approaches (based on themes emerging directly from the transcribed text). First, a team of six researchers reviewed the verbatim transcripts and grouped the data under two main categories—challenges and strategies. In the second stage of coding, major themes were developed, and verbatim text from transcripts was placed within these categories. We created, refined, or eliminated codes by establishing similarities and differences of the transcribed text. The codes were then grouped into major themes. Several steps were used to increase methodological rigor: (a) multiple coders participated in the analysis to ensure a wide range of viewpoints and discussions of varied perceptions were represented, (b) ambiguities and coding discrepancies were resolved by reviewing the focus group transcripts and reaching consensus, and (c) rival explanations were considered during analysis to facilitate trimming and validate our findings. Finally, to facilitate a cross walk between transition challenges and strategies, and to support the development of intervention programs, we categorized themes into four domains for intervention: (1) client/clinical, (2) family and natural supports, (3) ACT staff and team, and (4) public mental health system. The study was approved by the institutional review boards of New York State Psychiatric Institute and the New York State OMH. All authors certify responsibility for this study.

## Results

Results are presented in two sections. First we describe the themes that emerged around perceived challenges to transition (Table 1), and second we describe the themes that emerged around transition strategies (Table 2). Themes were grouped under four domains: (1) client/clinical, (2) family and natural supports, (3) ACT staff and team, and (4) public mental health system.

**Table 1:** Perceived challenges of transition voiced by ACT team leaders: themes and examples

Themes	Example
*Client and clinical challenges*	
Client resistance to transition	
Loss of relationships for clients	“They [staff] become almost family… they [clients] don’t want to step down and lose that person that understands them and gets them and has made their world a better place, they don’t want to let that person go.”
Clients’ fear of failure	“[For] this subset of people who’ve done very well with us… [and] have a life, stability in the community now for years, the idea that they would lose that stability I think is their fear.”
Client preference for ACT treatment approach	“… We offer choice. And that makes an amazing difference in the way they perceive their treatment that they don’t get elsewhere. You know, we don’t say, ‘do this.’ We say, ‘these are your options, what do you want to do?’ We don’t tell them what to do, and that is a lot of times why they don’t want to go anyplace else.”
Limited clinical expectations of success	
Relapse in the absence of ACT support	“… There are a lot of people that are really very functional in the community, have a job, have a really nice life… but if ACT lets them go they will never show up at a clinic… you have certain clients who are just going to be [ACT] lifers, for lack of a better word.”
Limited wellness management skills	“We’re not teaching our clients well enough that their illness is cyclical, about their triggers to relapse, how to identify [when] things are going bad and what to do… and that they don’t necessarily need the ACT team.”
*Family and natural supports related challenges*	
Loss of relationships for the family	“ACT is often the longest stable environment that a lot of [ACT clients] have been in. Whether or not they are necessarily psychiatrically stable through that whole experience, the families know that they [clients] are not going to get kicked out, they are not going to have to have a new therapist, you know (M3)… They [families] have become reliant (M1).”
Families’ limited expectations of success and fear of failure	“… They [families] perceive ACT as the one part of the system that hasn’t failed them. You know, you haven’t given up on my family member. You are the one part of the system that hasn’t fallen down on them. And so, in that respect, you can understand their concern, understand their anxiety about losing this lifeline.”
Family preference for ACT treatment approach	“And the families are extremely reluctant to give up the ACT team, and they don’t really see anything out there that is like there. So when we are talking about this we have to remember that there is no other service that provides what we are doing for the family and the home, and it is often helping other members of the family with stuff. And they won’t get that in a clinic.”
*ACT staff and team challenges*	
Maintaining a balanced case-load (high/low acuity)	“It’s really important for staff to see, on a day to day basis, people who have done well, who have stayed out of the hospital, who have stopped going to jail. Because the population that we target is so high need, you [need] that balance.”
Loss of relationships for ACT staff	“It’s a little bit about [a] parental type thing… you care about the person, you want them to do well, so you’re always working towards independence… but when it comes time for them to leave, it’s a loss there for us as well.”
*Public mental health system challenges*	
Access to community based mental health services	*Waiting Lists* “…in our county all the mental health clinics have a four to 6 month waiting list…and sometimes they cut off the list and say, ‘we can’t take anymore [clients]’.” *Transportation Barriers* “Our folks that are out in the rural counties, if they don’t have transportation and there’s no transportation to get them to their clinic appointments, they would need something in place to help them get to the clinic. I mean they’re not within walking distance, there [are] no buses, there [are] no cabs… so, we could really help someone adjust to a clinic, but then they can’t get there *Eligibility restrictions* “The most disabled [clients] are rejected you know, [if] they have a history of violence, hitting staff or fire setting…”
Stigma against ACT clients	“There’s some degree of stigma for the ACT clients in the community mental health setting. The fact that we target individuals who have failed, haven’t made it, or can’t engage in those traditional settings…since [clients] have been associated with ACT, there [is] this stigma.”

**Table 2:** Strategies voiced by ACT team leaders: themes and examples

Themes	Examples
*Client and clinical strategies*	
Building skills for transition	*Keeping office based appointments.* “… We have [clients] start coming in [to the office] once or twice a month [and] gradually work [with] them.” *Develop coping skills to manage daily stress.* “… Help them to develop problem-solving skills when they are encountering some normal life stresses.”
Celebrating success and new beginnings	“We’ve taken clients out to lunch with a couple other team members, and we acknowledge it as accomplishment but [without] too much pressure, you know, we always let them know that we’ll be following them officially for 90 days…”
*Family and natural supports related strategies*	
Educating and supporting natural supports	“… Incorporating skills for families to implement, because if they are going to be assuming more of a care giver kind of a role, at least in a increased capacity, that that would make them a little more comfortable in doing so, and would actually be a larger support for that person.”
Expanding supports and community resources	“A lot of [the work] is working on family and social relationships, improving communication…and learning to become familiar with community resources.” “If they were accessing the [natural supports] available in the community, they would need less support. But [since ACT has responded to crisis the way it does] we are going to have to un-teach them all of that, and teach them how to be more integrated into the community and to access what’s available.”
*ACT staff and team strategies*	
Integrating transition into routine of ACT services	*Orientation to time*-*limited services* “So I think the philosophy that you set in the beginning really dictates how you work, and I was told from the very beginning, we are not keeping them forever. So our philosophy has been, the minute you get them, start working on something, start teaching them something so that they are not dependent on you, and then they will assume that they have to do something, if you feel that they can’t do something then you teach them how to do it, you don’t do it for them.” *Integrating discharge planning into workflow* “… We’ve put some more structure around how we introduce [transition] to clients. Every month we review [the] treatment plans [that] are due the next month, and we talk about [transition]… [and we make a] team decision [regarding] who’s ready to step down, who’s not, and what that might look like for people.” *Incorporating ongoing assessment of transition into workflow.* “[Discharge planning is a] series of steps in which we try to get that person to where we think they should be in order to facilitate that discharge, which is something that we should do throughout, and that we do in most cases, in terms of planning for discharge. But one of things we realized is that it is really important to start discharge planning as soon as the person enters into the program… it makes it much easier to transition from that point.”
*Public mental health system*	
Enhanced coordination with new providers	“… If they’re going to an ICM… you have transition meetings and a lot of phone contact. And certainly going to a clinic is a sort of a bigger step down, then being transferred down to a different level of home visiting service… I would say that that would be an important thing for somebody in the team [to do] who has a knowledge of the client, and has a relationship most importantly I guess with the client. Being able to sort of hand that relationship over.”

### Perceived Challenges of Transition Voiced by ACT Team Leaders

#### Client and Clinical Challenges: Client Resistance to Transition

ACT team leaders anticipated that clients would not want to transition from ACT for a number of reasons including that clients would not want to give up the relationship with the ACT staff, that clients fear they would relapse in the absence of ACT support, and that clients prefer the ACT model to other service options.

##### Loss of Relationships for Clients

Participants discussed their perceptions of clients’ concerns around the loss of relationship with the team. Clinicians reported clients would be reluctant to break the strong bonds formed with ACT staff over the years. The team leaders felt that the ACT team had become a surrogate family for many clients, and the single most long lasting relationship some clients had experienced. Team leaders also expressed concern that clients had been told that ACT was “for life”, and had expectations that they would be able to continue on the team “forever.” They also reported that clients dislike the idea of “starting over” with new providers, and reviewing their personal and treatment histories with a new service provider.

##### Clients’ Fear of Failure

Team leaders thought that ACT clients attributed gains and continued stability to the ACT team, and feared a relapse in the absence of ACT supports. Team leaders anticipated that ACT clients would be hesitant to leave ACT due to concerns about loss of gains made while on the team, particularly those clients who had made a lot of progress since admission.

##### Client Preference for ACT Treatment Approach

Team leaders underscored the differences in treatment approach and philosophy between ACT and other mental health community based services. Participants described other community based services as more rigid than ACT, less assertive and engaging, and most often restricted to the office setting. ACT teams were viewed as placing more emphasis on person-centered treatment, i.e., working with clients based on their identified goals and preferred services. Team leaders believed that ACT clients recognized these differences and preferred the ACT treatment approach to other treatment options. Team leaders felt that clients may also have had negative experiences with other providers before coming to ACT that would make clients hesitant to return to these services. Finally team leaders felt the ACT approach was more effective than other services for the clients they served, and believed clients shared this view. They thought that clients believed the ACT model had helped them, while previous treatment services had not, and consequently would prefer to remain on ACT.

#### Client and Clinical Challenges: Limited Clinical Expectation of Success

Some team leaders expressed concern that transition would not be successful, due primarily to two overarching clinical concerns, that even very stable clients would relapse without ACT support, and that ACT clients did not have sufficient independence in wellness management and coping skills.

##### Relapse in the Absence of ACT Support

Participants expressed doubts around clients’ ability to sustain gains in the absence of supports provided by ACT. Across groups, team leaders described clients’ trajectory with ACT from the time of intake, a time of crisis, to later stability and successes. Team leaders focused on the gains clients had made with their team, and voiced concerns that clients would relapse without ACT.

##### Limited Wellness Management Skills

Team leaders identified clients’ limited skills to manage daily living activities and stress on their own as another barrier to transition. ACT teams underscored the supports they provided to clients and questioned whether ACT clients would be able to maintain stability without ongoing ACT support. Activities such as managing medications, accessing public transportation, socializing, and managing money, and the ability to recognize triggers and apply coping mechanisms were identified as potential vulnerabilities that could lead to destabilization and relapse. Some team leaders also suggested that ACT teams were not focusing on building skills for transition. They recognized that ACT supports may inadvertently foster dependence rather than promote independence, and that dependence upon ACT would need to be “unlearned.”

#### Family and Natural Supports Related Challenges

##### Loss of Relationships for the Family

Clinicians anticipated that clients’ relatives would be concerned about the loss of relationship with the ACT team as many have come to rely on the ACT team for support with responsibilities of care they had assumed in the past, and expected to assume again after discharge. Some team leaders suggested that the families were able to have more of a relationship with the ACT team than they had with previous providers, in part due to the in vivo nature of services, where ACT staff spent time in the family home.

##### Families’ Limited Expectations of Success and Fear of Failure

Participants believed that family members shared their concerns around potential crisis and relapse in the absence of the ACT team. Clinicians felt that families attributed clinical gains and stability to ACT services, and would have concerns about risking a loss of gains. Team leaders suggested that families would not see the client as well enough to succeed without ACT support.

##### Family Preference for ACT Treatment Approach

Team leaders believed that families were well aware of the difference between ACT and other services, and that families preferred the ACT approach particularly assertive outreach, no discharge orientation, in vivo services in the home, and family work. ACT staff anticipated that families may not be supportive of plans to discharge their family member due to these differences.

#### ACT Staff and Team Challenges

ACT team leaders identified some challenges they anticipated for themselves and their staff in transitioning clients, including impact on caseload, and staff challenges in ending relationships with long-term clients.

##### Maintaining a Balanced Caseload

Team leaders anticipated challenges resulting from discharging clients who had made significant progress in their recovery, and filling vacancies with new clients with acute needs. This shift in caseload was expected to generate more work for the team, requiring more frequency and longer duration of clinical contacts. In addition, team leaders noted that the ability to continue working with clients who were doing well had positive effects on staff.

##### Loss of Relationships for ACT Staff

Team leaders reported their own ambivalence about transition, stemming from a loss of relationship with the client. Participants referred to their role as a client’s “family”, and as such, discharge represented more than an end to the therapeutic relationship. The word “family” was used by all focus groups to refer to the ACT “family” rather than to clients’ actual family.

#### Public Mental Health System Challenges

##### Access to Community Based Mental Health Services

Team leaders described several challenges with respect to accessing other mental health services for transitioning clients. These included waiting lists, transportation barriers, and restrictive admission criteria. These barriers varied by region. Participants in the New York City region described long delays in the transition process because clients are placed on waiting lists for clinic or case management services. Transportation barriers were particularly concerning for rural areas of the state where travel to a clinic might be 45 miles or more without public transportation options. In addition, some teams expressed concerns about restrictions placed by some service providers on admissions. For example, some had experienced clinics’ refusal to admit clients with a history of violence or an active substance abuse problem.

##### Stigma Against ACT Clients

Participants perceived stigma by some mental health providers towards ACT clients as a barrier to transition. During referral attempts some providers raised questions around clients’ readiness for transition or were reluctant to proceed with intake. In addition, participants believed clients may be reluctant to transition based on having experienced stigma in other mental health service settings. Clients may also have stigmatized views of mental health clinics. One participant shared that a client described the clinic as a place where ‘crazy people go’ and contrasted this with ACT services where contact with providers is in a person’s own home or community.

### Transition Strategies Voiced by ACT Team Leaders

#### Client and Clinical Strategies

##### Building Skills for Transition

Participants emphasized the importance of preparing clients for transition to less intensive services. Preparation typically involved educating clients to become more independent, especially with regard to attending office visits, identified as an important skill for success in community based mental health services. Participants explained that preparation should include strategies focused on managing stresses of daily life (e.g., waiting for an appointment).

##### Celebrating Success and New Beginnings

Nearly all participants described ways in which teams could celebrate the success of clients who were transitioning from ACT to less intensive services. Activities included arranging graduation ceremonies, events, parties, and celebrating with food. Team leaders had tried out celebrations and found them to be an important part of the transition process. Celebrations signify for the client (and team) that the client had made significant progress. When celebrations involved other clients, they may help to model success for other clients.

#### Family and Natural Supports

##### Educating and Engaging Natural Supports

Participants identified education and support of clients’ family members, friends and other supportive people in their lives as a critical element in facilitating transitions. This involved engaging natural supports early in the transition process by educating them about the steps to transition, the progress their loved one has made while in ACT, and the opportunities for continued growth. Team leaders suggested that ACT should work with families to build and practice skills to support their family member during and after transition.

##### Expanding Supports and Community Resources

Another key ingredient in preparation was helping clients to build and expand supportive relationships not only with immediate family but with friends, landlords, employers, etc. They described the importance of increasing client access and engagement with existing and new community resources that could be tapped into to prevent crisis or accessed in the event of a relapse.

#### ACT Staff and Team Strategies

##### Integrating Transition Into Routine ACT Services

The most prominent team-level strategy suggested was to structure the transition process, though few teams had a structure in place. Participants described multiple strategies to better facilitate the transition process, including establishing expectations of graduation at intake, ongoing assessment of transition readiness, and developing and implementing a transition plan for clients.

Team leaders reported that clients who were introduced to transition earlier in treatment were more open to transition than clients who were introduced to transition later in treatment.

Although teams lacked formal criteria or assessment for transition, most participants described the importance of assessing clients’ readiness for transition. The areas that were the most prominently discussed across the six focus groups included (1) stable housing, (2) ability to live independently with little assistance with regard to cooking meals, managing money, cleaning, and shopping, (3) no hospitalizations or incarcerations in the past year, (4) access to natural supports (i.e., family and friends), (5) being employed or working towards vocational goals (e.g., securing a job, participating in job training programs), (6) ability to attend office-based visits, and (7) ability to manage medications independently. Participants discussed the importance of preparing clients for transition through planning for the areas of need identified above. They explained that it was critical to systematically assess clients’ level of independence in these areas and incorporate into plans for clients to help build skills for transition. Although ACT teams routinely worked on these areas, it was with the assumption of ongoing ACT support, rather teaching clients how to manage these domains with a lower level of service.

#### Public Mental Health System

##### Enhanced Coordination with New Providers

Participants perceived coordination of care as a key element in transition, and shared strategies they had used to ensure continuity of care, such as pre-transition meetings and multiple phone contacts with new providers. They felt that the more opportunities clients had to meet their new providers before transition, the more comfortable they were at the time of transfer. Other strategies to support continuity of care included having a dedicated “transition case manager” to serve as a bridger between ACT and the new services.

## Discussion

Transition from ACT to less intensive services is a vulnerable period where there is both some risk of loss of gains made during ACT, but for most an opportunity to move toward greater independence (Rosenheck and Dennis 2001; Rosenheck et al. 2010; Hackman and Stowell 2009). This study sheds light on the challenges and strategies of transition from the perspective of team leaders for the following domains: (1) client/clinical, (2) family and natural supports, (3) ACT staff and team, (4) public mental health system.

### Client and Clinical Domain

Several themes emerged related to client and clinical concerns about discharge from ACT. ACT team leaders felt strongly that clients would be resistant to transition from ACT based on a preference for ACT services, fear of relapse, and loss of relationship with ACT team. It is important to note that ACT team leaders do not speak for clients, and client attitudes about transition need to be assessed directly from ACT clients as an area for future research. However, the strength of clinicians’ beliefs about client resistance represent a clinical challenge.

Clinicians had concerns that clients would not retain gains made on ACT, and that clients did not have sufficient self-management and coping skills. Clinicians perceive that clients’ success in ACT is due in part to the high intensity of ACT services. ACT is conceptually and philosophically different from other community based mental health services. ACT services are assertive and flexible and provided directly to clients in their community or home, while standard case management services are office-based and brokered (Bond et al. 2001). In this study, some clinicians believed that once clients transition from ACT services to less intensive services, they become more vulnerable to relapse and hospitalization. This reaction may be the result of the “clinician’s illusion” that is, clinicians who see clients only when they are ill may draw conclusions that they will always be ill (Cohen and Cohen 1984). ACT staff in particular may be less inclined to see recovery in clients given the high severity of need and functional impairment of their clients. At the same time, the “no discharge” policy stems from the initial ACT study which found that clients deteriorated following withdrawal of ACT services after 1 year (Stein and Test 1980). Since then, however, ACT services have evolved with several studies confirming that although relapse is a risk, for select clients successful transition is the norm (Rosenheck et al. 2010; Rosenheck and Dennis 2001; Hackman and Stowell 2009).

Participants described several strategies to address clinical concerns that are useful to consider in future research and practice. First, clinicians described the importance of engaging clients in transition and preparing for lower levels of service. ACT team leaders suggested that clients should be evaluated for transition, and develop transition focused treatment plans to build skills needed to succeed at a lower level of care. Although existing ACT treatment plans may address many of the same clinical and functional areas, they assume ongoing support of the team, rather than building and testing skills to promote independence from the team. For example, team leaders identified practicing attending scheduled office-based appointments, and the associated skills, including scheduling appointments, transportation to appointments, arriving on time, and coping with frustrations while waiting to be seen. This shift in orientation may help clinicians to identify and minimize interventions that inadvertently promote dependency.

The proposed approach to transition treatment planning, and building and testing skills for success at a lower level of care is reminiscent of Critical Time Intervention (CTI), a time limited model of case management (Herman et al. 2007). The CTI model involves a structured and phased approach to case management focusing on successful engagement, decreasing recidivism, and improving outcomes in a lower level of care. The three phases of CTI include, transition (developing and implementing a transition plan, and providing specialized services), try-out (facilitate and test clients problem solving skills) and transfer of care (terminate CTI services with support network in place) (Herman et al. 2007). CTI was originally developed to help support the transition from homeless to housed, but has been adapted for discharge from hospitals to lower levels of service (Dixon et al. 2009), and jails to community (Draine and Herman 2007). Adapting CTI for ACT, either as a time-limited approach to delivering ACT services, or as a time-limited case management program for ACT clients to transition to, may be a promising area for future research.

An additional strategy with broad support from team leaders was to celebrate graduation from ACT. Celebrations may help to assign positive meaning to the end of ACT treatment. Involvement of other ACT clients in a graduation event gives the ACT graduate the opportunity to be a peer role model for successful transition. Importantly, celebrations may also help to address a challenged raised by ACT team leaders concerning clients, family and staff fears of relapse. Team leaders suggested that these concerns were based in part upon beliefs that the client stability depended upon ongoing ACT support. Not only were gains attributed to ACT, but sustained stability was also attributed to ACT, rather than an understanding that the client had made progress, and is now ready to transition to a lower level of care. Celebrations of success may help indicate that it is the client’s achievement, and help reassign attribution of sustained gains to the client.

### Family and Natural Supports

Family related challenges identified by ACT team leaders included an anticipation of family resistance to transition due to family preference for ACT approach, family fears of relapse, and loss of relationship with ACT team. As with clients, ACT team leaders cannot speak for families; families’ views need to be assessed from family members of ACT clients as an area of future study. However, team leaders’ views of family concerns represent a barrier to discharge and a clinical challenge for ACT teams. Strategies suggested to support family engagement in the transition process include early involvement of families, education, transition planning with families, and building and testing families’ skills to support transition. Working with families is considered an element of ACT fidelity (Teague et al. 1998), but has been considered a low performance area (Bond et al. 2001). Critical Time Intervention underscores the importance in involving long term supports, including families, in all phases including transition planning and service delivery, try-out phase, and transfer of care (Herman et al. 2007).

### ACT Staff and Team

A prominent theme from this study is the ambivalence about ACT transition among clinicians. This ambivalence stemmed from different sources. The ACT relationship was a key factor that influenced clinicians’ ambivalent feelings towards transition, and their perceptions of clients’ and families’ ambivalence. Although feelings of ambivalence and sadness are often normal reactions to terminations, the nature and intensity of ACT services, including the traditional “no discharge” philosophy, may intensify this struggle. The high intensity of ACT services may blur boundaries of the ACT relationship (Angell and Mahoney 2007). Teams that have not had planned discharge as a treatment goal for their clients may also be out of practice in dealing with termination issues. Strategies to consider are incorporating staff support and clinical supervision around termination issues.

ACT team leaders also expressed concerns about the impact of transitions on the caseload balance. Transition work is time consuming in itself, but after a stable client is discharged the team receives a new referral with acute needs. In addition team leaders spoke of the impact of having successful clients remain on the team, as a benefit to team morale and a reminder to staff of the benefits of their work.

The strategies proposed by team leaders to support the ACT team focused on the need for more structure to the transition process such that it is fully integrated into the routine workflow of ACT services. ACT teams historically had a “no discharge policy” orientation, and may lack experience with discharge practices, awareness of community service options, and guidelines for transitioning clients to less intensive services. Strategies that clinicians suggested to better integrate transition into the routine of ACT services included providing clients with an orientation to transition at admission so that it is an expected goal of treatment, and developing a series of steps to the transition process that include an assessment of transition readiness and transition planning.

### Public Mental Health System

Clinicians described system level challenges which delayed or prevented transfer to other services such as access to transportation, waiting lists, and eligibility or selection criteria of referral programs. Clinicians also expressed concern about the hesitation of new providers to accept referrals from ACT, which they attributed to stigma associated with being an ACT client. It may be that new providers’ hesitation is a generalization based on past experience of working with an ACT client who did not respond well to other types of treatment. On the other hand, new providers may be hesitant to accept ACT clients because they lack information about the progress the client has made while receiving ACT services. Clinicians described the importance of coordinating care with new providers to ensure continuity across levels of care. Enhanced coordination of services with new providers, including ongoing communication with the new provider and face to face pre-transition planning meetings can be used to both support clients and discuss their history and progress with the new provider.

### Limitations and Conclusions

This study has several limitations. The sample was restricted to ACT team leaders in New York State which may limit generalizability of findings to other states or high intensity program types. However, the focus groups included clinicians in every region of the State, including urban, rural and suburban areas to allow for a description of challenges and strategies in diverse settings. Future research is needed to explore ACT teams in other states, and to examine challenges and strategies pre- and post-transition, since the concerns identified may change. In addition, it is important to elicit challenges and strategies of transition from the perspectives of current and former ACT clients, family members, as well as clinicians in other community based mental health services who work with former ACT clients.

Despite these limitations, this study provides important insights about the challenges and strategies of transition from ACT to less intensive services. The time-unlimited nature of ACT may conflict with recovery-oriented, person-centered care that is now at the forefront of mental health policy and services. There is increasing concern that time-unlimited services may restrict access to those in greatest need of ACT services. Many states, including New York State, have made considerable financial investment in ACT services because it provides specialized services for persons with severe mental illness. While there is evidence that ACT is cost effective, especially for persons with high hospital use prior to ACT (Essock et al. 1998; Morrissey et al. 2013; Slade et al. 2013) a recent study on Washington State PACT programs by Domino et al. (2013) found that the largest reductions in hospital costs are observed early on after PACT enrollment but taper off over time. This finding suggests that transition to lower levels of care may be indicated as clients become more stable over time, and a more cost effective approach for those who can sustain clinical gains. The results from this study highlight the complexity and challenges of discharging ACT clients to lower levels of care, but also suggest strategies for addressing these challenges and supporting transitions. This information can be used to inform the development of clinical models that effectively support the transition process.
